# The Long Noncoding Transcript HNSCAT1 Activates KRT80 and Triggers Therapeutic Efficacy in Head and Neck Squamous Cell Carcinoma

**DOI:** 10.1155/2022/4156966

**Published:** 2022-08-04

**Authors:** Yixuan Zhao, Xin Huang, Zewei Zhang, Haizhou Li, Tao Zan

**Affiliations:** ^1^Department of Plastic and Reconstructive Surgery, Ninth People's Hospital, Shanghai Jiao Tong University School of Medicine, Shanghai, China; ^2^Department of Plastic and Reconstructive Surgery, First Affiliated Hospital of Zhengzhou University, Zhengzhou, Henan Province, China

## Abstract

Head and neck squamous carcinoma (HNSC) is the most prevalent malignancy of the head and neck regions. Long noncoding RNAs (lncRNAs) are vital in tumorigenesis regulation. However, the role of lncRNAs in HNSC requires further exploration. Herein, through bioinformatic assays using The Cancer Genome Atlas (TCGA) datasets, rapid amplification of cDNA ends (RACE) assays, and RNA-FISH, we revealed that a novel cytoplasmic transcript, HNSC-associated transcript 1 (HNSCAT1, previously recognized as linc01269), was downregulated in tumor samples and advanced tumor stages and was also associated with favorable outcomes in HNSC. Overexpression of HNSCAT1 triggered treatment efficacy in HNSCs both *in vivo* and *in vitro*. More importantly, through high-throughput transcriptome analysis (RNA-seq, in NODE database, OEZ007550), we identified KRT80, a tumor suppressor in HNSC, as the target of HNSCAT1. KRT80 expression was modulated by lncRNA HNSCAT1 and presented a positive correlation in tumor samples (*R* = 0.52, *p* < 0.001). Intriguingly, we identified that miR-1245 simultaneously interacts with KRT80 and HNSCAT1, which bridges the regulatory function between KRT80 and HNSCAT1. Conclusively, our study demonstrated that lncRNA HNSCAT1 functions as a necessary tumor inhibitor in HNSC, which provides a novel mechanism of lncRNA function and provides alternative targets for the diagnosis and treatment of HNSC.

## 1. Introduction

Head and neck squamous cell carcinomas (HNSCs) are the most frequent malignancies of the head and neck regions and are a heterogeneous group of tumor entities covering several anatomical subsites in the head and neck [1]. Approximately half a million patients are diagnosed annually with HNSC, and it is a major contributor to cancer-related mortality worldwide [2]. The pathogenesis of HNSC is multifactorial [3, 4], including (a) an acute demand for protein synthesis that is often a result of oncogene activation, (b) inadequate oxygen and nutrient supply as well as chemo/radiotherapy exposure, and (c) immune surveillance escape [5–7].

Studies are aimed at identifying biomarkers for stratifying patients into clinically significant groups and for the development of effective targeted therapies [8]. However, the limited therapeutic response and aggressive property of HNSCs are affected by the complex deviations in intracellular signaling pathways and by extracellular microenvironment behaviors [9, 10]. The tumor landscape of HNSC permits their aggressive nature, which is enhanced by immune system activity, tumor hypoxia, and microbiome effects [11, 12]. Because of the complexity of tumor onset and the limited response to current therapy, it is important to determine the mechanism of oncogenesis of HNSC.

Risk factors for HNSC include human papillomavirus infections, tobacco and alcohol intake, and aberrant genetic as well as epigenetic alterations. For example, NOTCH, EGFR, and MET modifications enhance cell proliferation, migration, and survival via PI3K, RAS/RAF/ERK, and JAK/STAT signaling, and these pathways are frequently deregulated in HNSC [13]. p53 pathway disruption is associated with increased genomic instability [14]. Moreover, loss of epigenetic balance has been proven to be a driving event for the initiation and progression of HNSC [15, 16]. For example, CDKN2A, a tumor suppressor gene, suppresses cyclin-dependent kinase expression as well as cell cycle progression. In HNSC cells, many promoters of tumor suppressors are often hypermethylated, contributing to the abnormal silencing of these tumor-inhibiting genes [17, 18]. More importantly, long noncoding RNAs, which are transcripts (>200 nt) without coding capacity, have been proven to be associated with the pathogenesis of HNSC [19]. For instance, the lncRNA MIR31HG targets P21 and HIF1A to mediate head and neck cancer cell proliferation and tumorigenesis by stimulating cell cycle progression [20]. Moreover, an IFN*α*-initiated long noncoding RNA, lncMX1-215, exerts negative effects on immune suppression by interrupting the acetylation of H3K27 in HNSC [21]. However, the role of linc01269, a novel transcript with an unknown function, in the progression of HNSC remains to be further explored.

We thus aimed to determine the significance of abnormal lncRNA expression in the tumorigenesis of HNSC. Through bioinformatic assays and RACE assays, we found that a novel transcript, HNSCAT1 (previously identified as linc01269), was downregulated in HNSC and associated with favorable outcomes in the TCGA cohort. Moreover, overexpression of HNSCAT1 significantly inhibited tumor proliferation and migration *in vitro* and *in vivo*. Mechanistically, HNSCAT1 interacts with miRNA-1245 and promotes KRT80 expression. Our study elucidates the molecular biology of HNSCs and provides a basis for further studies on HNSC diagnosis and treatment.

## 2. Materials and Methods

### 2.1. Cell Culture

Primary keratinocytes were collected from eyelids during blepharoplasty. Specimens were washed in Dulbecco's phosphate-buffered saline containing 20 *μ*g/mL gentamicin. The samples were cut into 5 mm^3^ pieces, and the epidermis was isolated. Dispase (Roche) solution in Dulbecco's phosphate buffered saline with 5 *μ*g/mL gentamicin was applied to the specimen pieces for 12 hours at 4°C. The epidermis was carefully separated and placed into a solution containing 0.05% trypsin-EDTA at 37°C for 15 minutes. The cells were then cultured in defined keratinocyte serum-free medium (Gibco). HaCaT, SCL-1, Cal27, and Colo16 cells were purchased from ATCC and cultured in DMEM (Gibco) supplemented with 10% fetal bovine serum (FBS; Gibco), streptomycin (100 mg/mL), and penicillin (100 U/mL). Incubation was performed at 37°C in a 5% CO_2_ humid atmosphere. This study obtained ethical approval from the Independent Ethics Committee of Shanghai Ninth People's Hospital (registration no. 2015(72)).

### 2.2. Plasmid Construction and Rapid Amplification of cDNA Ends

pLKO.1, pCDH, and pCMV were used in our study. ShRNA sequences were generated by PCR and then cloned into the pLKO.1 vector. The KRT80 and lncHNSCAT1 overexpression cassette was generated by PCR, cloned into the pCDH vector, and verified by DNA sequencing. A rapid amplification of cDNA ends (RACE) assay was carried out with the 5′ RACE System for Rapid Amplification of cDNA Ends (Invitrogen, #18374-058) and the 3′ RACE System for Rapid Amplification of cDNA Ends (Invitrogen, #18373-019) according to the manufacturer's protocols. The amplified DNA fragment was cloned into the pGEM-T Easy vector and validated by Sanger sequencing (Sangon Biotech, China).

### 2.3. CCK-8, Colony Formation, and Wound Healing Assays

Cell proliferation was assessed using CCK-8 assays (HY-K0301, MCE) according to the manufacturer's instructions. Briefly, cell seeding (3000 cells/100 mL) was performed in triplicate in 96-well plates. At various time points, the dye solution was added, followed by incubation at 37°C for 4 h. The absorbance was measured at 570 nm. For the colony formation assay, 1 mL of complete medium with 1,000 cells was added to each well of a six-well plate. After 1–2 weeks, the plate was stained using crystal violet (0.25%). A wound healing assay was performed after seeding 500,000 cells into a 6-well plate. A wound was made by manually scraping the cell monolayer with a 200 *μ*l pipet tip. Images were taken at the indicated times. The percentage wound healing was calculated using the formula: wound healing = (initial wound area − unhealed wound area)/initial wound area × 100%.

### 2.4. Subcutaneous Xenograft Study

The use of animals in this study was permitted by the Shanghai Jiao Tong University Animal Care and Use Committee. Experiments were performed following animal policies of Shanghai Jiao Tong University and the National Health and Family Planning Commission of China guidelines. Cell harvesting was performed by trypsinization, after which the cells were washed twice using PBS (GIBCO). Male BALB/c nude mice (aged 4 weeks) were used as models for experiments. Approximately 1 × 10^6^ HNSC cells from each group were subcutaneously injected into the underarm region of the mice. At 14 days postinjection, the mice were euthanized and sacrificed for tumor resection. The formula *V* = *ab*^2^/2 was used to determine tumor volumes, with *a* and *b* denoting tumor length and width, respectively.

### 2.5. RNA-seq

The EZ-press RNA Purification Kit (B0004) was used for extraction of total RNA, whose integrity was confirmed using a 2100 Bioanalyzer (Agilent Technologies, USA). RNA concentrations were assessed using a Qubit 2.0 fluorometer and a Qubit RNA Assay Kit (Life Technologies, Carlsbad, CA, USA). Using total RNA (100 ng) and the Illumina TruSeq RNA Sample Prep Kit (San Diego, CA, USA) as instructed by the manufacturer, libraries were prepared. Sequencing and basic informatic assays were conducted by Kangcheng Biotech, Inc. (Shanghai, China).

### 2.6. Western Blotting

Cells were harvested and rinsed with PBS. Lysis buffer (RIPA) was used to prepare cell extracts, which were centrifuged for 10 min at 12,000 × g. Protein samples were separated by SDS–PAGE (7.5%). The proteins were transferred to PVDF membranes, which were blocked at room temperature (RT) for 1 h using 5% BSA followed by overnight incubation in the presence of 2.0 *μ*g/mL antibody in 5% BSA at 4°C. Anti-GAPDH (#60004-1-Ig, 1 : 5000 dilution, Proteintech) and anti-KRT80 (1 : 500 dilution, Abcam) were used. The unmodified images are shown in the supplementary figures (available here).

### 2.7. Biotin-Labeled RNA Pulldown

A lncHNSCAT1 targeting biotin-labeled probe (miR-1245-biotin) as well as a random oligo probe (RiboBio, Guangzhou, China) was incubated for 4 h with streptavidin Dynabeads (M280, Invitrogen, USA) at 4°C. Overnight incubation of HNSC cell lines was performed in the presence of bead/probe complexes at 4°C. Washing of the lncRNA/miRNA/bead complexes was performed twice followed by elution from beads. Then, HNSCAT1 and associated miRNA enrichments in the complexes were assessed by qRT–PCR.

### 2.8. Luciferase Assay

Transfection of cells in 6-well plates was performed using a pGL3-based luciferase vector fused or not fused to mutated or wild-type KRT80-3′UTR. Quantification of the efficiency of transfection was performed by cotransfection with an actin promoter-mediated Renilla luciferase reporter. Firefly and Renilla luciferase activities in every well were determined by a dual-luciferase reporter assay system (Promega). Relative luciferase activities of the KRT80-3′UTR plasmid were normalized to signals in firefly luciferase vector control-transfected cells under the same conditions.

### 2.9. Quantitative Real-Time PCR

RNA expression was analyzed by quantitative real-time PCR with Power SYBR Green PCR Master Mix (Applied Biosystems). This assay was performed on an ABI Prism 7500 sequence detector (Applied Biosystems). The primers for lncRNA and KRT80 are listed in Supplementary Table 1. All data were normalized to *β*-actin. All assays were run in triplicate. The 10 *μ*l samples contained 5 *μ*l Power SYBR Green PCR Master Mix (Applied Biosystems), 5 pmol of each primer, 3 *μ*l diethyl pyrocarbonate-treated water, and 1 *μ*l DNA. All assays were run in triplicate. The cycling parameters consisted of 95°C for 10 min, followed by 40 cycles of 95°C for 15 s, 60°C for 1 min, and 72°C for 30 s. These primers included lncHNSCAT1 (forward: GTGGCCCTTGATTGAGCTTG and reverse: TCATGGTGCTCTGTCCCTCA) and KRT80 (forward: GGACCTGGATGCAGAGTGTC and reverse: CAGCTGGCTCCGAGAGTATG).

### 2.10. RNA FISH

Briefly, cells were fixed with 4% formaldehyde/10% acetic acid and stored overnight in 70% ethanol. The fluorescence-labeled single-strand probes were synthesized (lncRNA HNSCAT1: TCATGGTGCTCTGTCCCTCA-Cy3) and then hybridized. 18S and U6 oligos were purchased from Ribo-bio (Shanghai). To increase the stability of RNA foci, RNA signals were detected with a tyramide-Alexa Fluor 546 signal amplification kit (Invitrogen). After labeling, fluorescence signals were detected using a microscope (BX41; Olympus).

### 2.11. Nuclear-Cytoplasmic Extraction

The cellular fraction was isolated as previously described [22]. In brief, 3 × 10^6^ cells were harvested and resuspended in DEPC-PBS. The cell pellets were then resuspended in 2 ml of hypotonic buffer (10 mM Tris–HCl (pH 7.4), 20 mM MgCl_2_, and 4% Triton X-100), and the cells were gently ground by a Dounce homogenizer and incubated for 30 min on ice. Then, the cells were centrifuged for 15 min at 3000 rpm; the supernatant containing the cytoplasmic component and the pellet containing the nuclear fraction were kept for RNA extraction.

### 2.12. Statistical Analysis

We performed the statistical analyses with the GraphPad Prism 9 software. Data are presented as the mean ± SD or SEM. The differences between two groups were calculated by unpaired two-tailed Student's *t*-test as indicated. Survival plots were generated by Kaplan–Meier curves, and *p* values were calculated by the log-rank test. Biological triplicates were assessed where indicated. *p* < 0.05 was considered to indicate statistical significance (^∗^*p* < 0.05, ^∗∗^*p* < 0.01, and ^∗∗∗^*p* < 0.001).

## 3. Results

### 3.1. HNSCAT1 Expression Is Decreased in HNSC, and This Downregulation Is Associated with a Favorable Outcome

**Figure 1:**
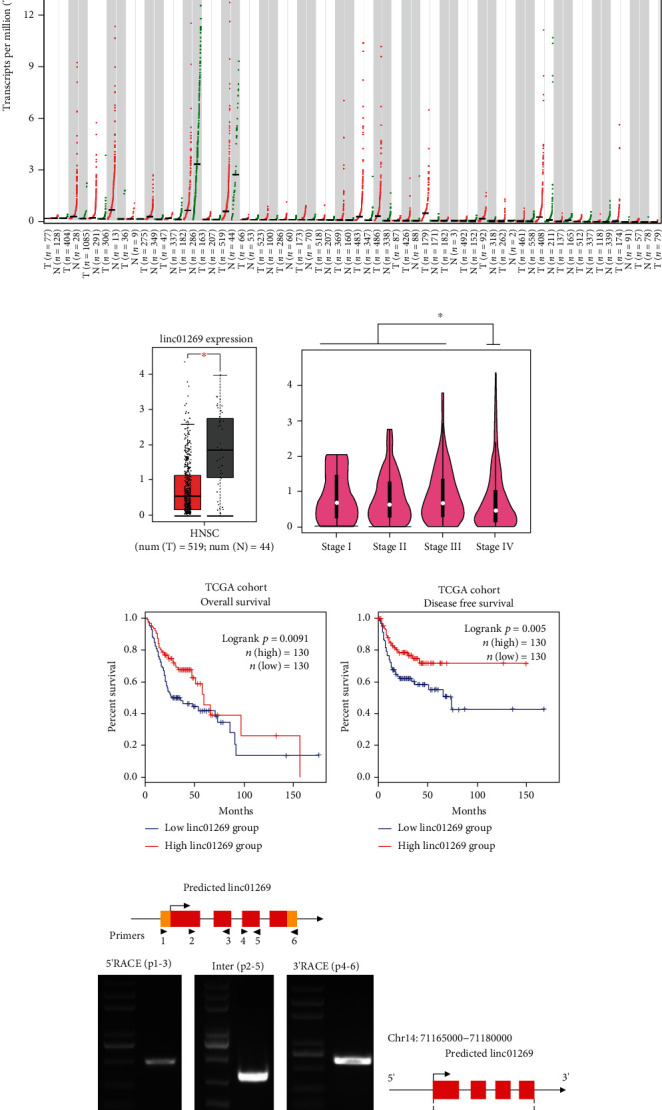
HNSCAT1 is downregulated in HNSC. (a) Pancancer analysis of RNA expression of linc01269 in TCGA database. (b) Expression of linc01269 in HNSC and normal samples. These data were acquired from GEPIA2 (http://gepia2.cancer-pku.cn). Significance was assessed by unpaired two-tailed Student's *t*-test. ^∗^*p* < 0.05. (c) Stage IV HNSC samples presented the lowest linc01269 expression level. The figure was generated on the GEPIA2 website (http://gepia2.cancer-pku.cn). Significance was assessed by unpaired two-tailed Student's *t*-test. ^∗^*p* < 0.05. (d, e) Elevated linc01269 expression was associated with favorable outcomes in terms of both (d) overall survival (log-rank *p* = 0.0091) and (e) disease-free survival (log-rank *p* = 0.005). (f, g) RACE assay for the identification of full-length linc01269. The novel transcript harbors a 166 bp extension in the 5′ terminus and a 303 bp extension in the 3′ terminus.

To investigate potential functional noncoding transcripts in HNSC, we queried TCGA dataset for expression analysis. Using GEPIA (http://gepia.cancer-pku.cn/), we identified the 50 most downregulated genes (Figure S1). Among these transcripts, we found that linc01269 was one of the most downregulated lncRNAs in HNSC samples (|log2FC| = 2.6, *p* < 0.001). Through pancancer analysis, we further confirmed that linc01269 was remarkably downregulated in HNSC and esophageal carcinoma (ESCA), while other malignancies remained unchanged (Figures 1(a) and 1(b), Figure S2A). Additionally, compared to the earlier stage of HNSC (I, II, and III), the late stage (IV) of HNSC presented with decreased expression of linc01269 (Figure 1(c)). Most importantly, elevated expression of linc01269 was associated with better outcome trends, including overall survival (OS, Figure 1(d)) and disease-free survival (DFS, Figure 1(e)). As the boundary of the long noncoding transcript is highly flexible [23], we then assessed whether *linc01269* encodes a new transcript in HNSC cells. Based on the National Center for Biotechnology Information (NCBI) database, linc*01269* lncRNA is 602 bp in length and has four exons. However, through rapid amplification of cDNA ends (RACE), we detected a novel isoform with different 5′- and 3′- boundaries of linc01269 (Figure 1(f)). This novel transcript was also identified to lack coding capacity (Figure S2B, coding potential score = −1.19). Specifically, exons 2 and 3 were consistent with predicted exons; however, exon 1 had an extra 166 bp fragment at its 5′ terminus, while exon 4 was extended by 303 bp. Compared to the predicted sequence, this transcript had an extra poly-A tail at its 3′ terminus (Figure 1(g), Figure S2C). These findings imply that the *linc01269* lncRNA isoform is a novel noncoding transcript in HNSC tumors; therefore, we named it *HNSC-associated transcript 1* (*HNSCAT1*).

### 3.2. Overexpression of *HNSCAT1* Attenuates HNSCs *In Vitro* and *In Vivo*

**Figure 2:**
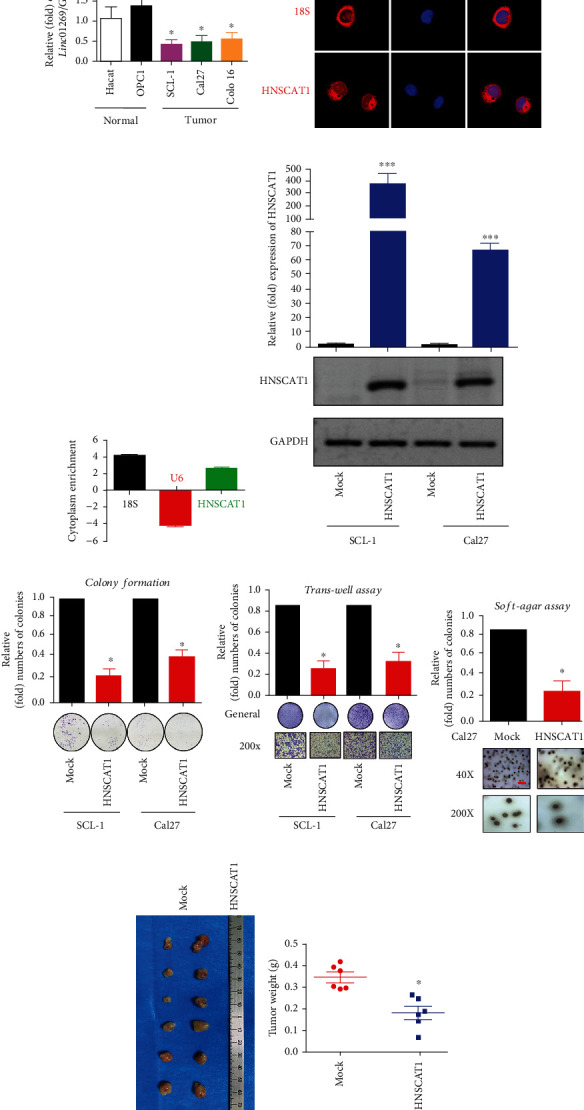
HNSCAT1 serves as a negative regulator of HNSC. (a) Real-time PCR revealed that lincRNA HNSCAT1 was downregulated in HNSC cell lines. HaCaT cells and primary keratinocytes (PK) served as normal controls. The value of HaCaT was set to 1. Data are presented as the means ± SD of three biological replicates. Significance was assessed by unpaired two-tailed Student's *t*-test. ^∗^*p* < 0.05. (b) RNA-FISH indicated that HNSCAT1 RNA was mainly distributed in the cytoplasm. (c) A nuclear-cytoplasmic RNA extraction assay was performed. Real-time PCR was performed to identify RNA distribution. U6 served as the nuclear control, while 18S was the cytoplasmic control. (d) Real-time PCR was performed to examine the overexpression efficacy of HNSCAT1 in SCL-1 and Cal27 cells. Data are presented as the means ± SD of three biological replicates. Significance was assessed by unpaired two-tailed Student's *t*-test. ^∗∗∗^*p* < 0.001. (e) Colony formation assays were conducted to determine proliferative capacity after overexpression of lincRNA HNSCAT1 in SCL-1 and Cal27 cells. Data are presented as the means ± SD of three biological replicates. Significance was assessed by unpaired two-tailed Student's *t*-test. ^∗^*p* < 0.05. (f) Transwell assays showed that migration was impaired after the restoration of lincRNA HNSCAT1 in SCL-1 and Cal27 cells. Data are presented as the means ± SD of three biological replicates. Significance was assessed by unpaired two-tailed Student's *t*-test. ^∗^*p* < 0.05. (g) A soft-agar colony formation assay was conducted to determine colony formation capacity after overexpression of lincRNA HNSCAT1 in Cal27 cells. Data are presented as the means ± SD of three biological replicates. Significance was assessed by unpaired two-tailed Student's *t*-test. ^∗^*p* < 0.05. (h) Subcutaneous xenografts were established in HNSCAT1-overexpressing and control cells. *N* = 6 for each group. (i) Tumor weight in each xenograft. ^∗^*p* < 0.05. Experiments were conducted in triplicate, and the results are shown as the mean ± SEM. ^∗^*p* < 0.05.

To assess whether this novel *HNSCAT1* was able to alter tumor behaviors, we first assessed lncHNSCAT1 expression levels in HNSC cell lines. Consistent with its expression level in TCGA samples, HNSCAT1 was significantly downregulated in HNSC cell lines, including SCL-1, Cal27, and Colo16 (Figure 2(a)), compared to normal keratinocyte cell lines (HaCaT) and primary keratinocytes (PKs). Through RNA-FISH and nuclear-cytoplasm separation assays, we found that HNSCAT1 was mainly distributed in the cytoplasm. In this assay, 18S RNA served as a cytoplasmic control, and U6 served as a nuclear RNA marker (Figures 2(b) and 2(c)). We further restored HNSCAT1 expression in HNSC cells by cloning full-length HNSCAT1 into an overexpression vector (Figure 2(d)). As expected, HNSCAT1-overexpressing HNSC cells presented an attenuated proliferation rate (Figure S3) and formed smaller and fewer colonies (Figure 2(e)). Transwell assays (Figure 2(f)) and wound healing assays (Figure S4A-D) demonstrated that HNSCAT1 overexpression resulted in impaired migration ability in HNSC cells. Moreover, a soft agar assay showed that tumorigenic capacity was inhibited after the restoration of HNSCAT1 expression (Figure 2(g)). Most importantly, in a xenograft mouse model, HNSCAT1-overexpressing cells formed smaller tumors, with a significant reduction in both size (Figure 2(h)) and weight (Figure 2(i)).

### 3.3. KRT80 Serves as a Downstream Target of lncRNA HNSCAT1

**Figure 3:**
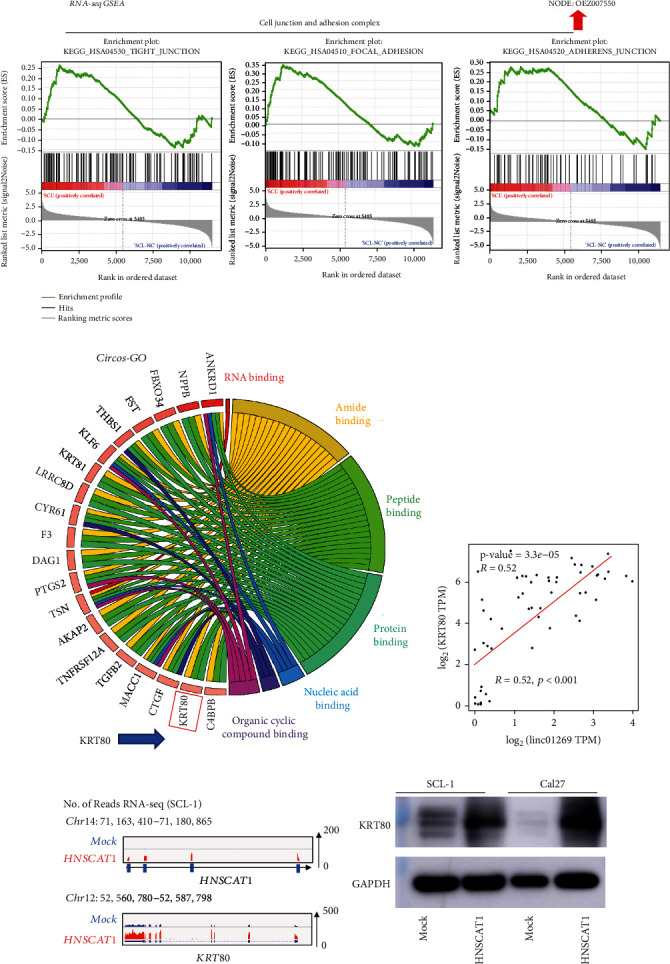
KRT80 was upregulated after overexpression of HNSCAT1. (a) GSEA was performed based on RNA-seq results after overexpression of HNSCAT1. Notably, cell junction- and adhesion complex-related signaling pathways were significantly upregulated in HNSCAT1-overexpressing cells. (b) A Circos analysis was performed and revealed that KRT80 mRNA expression was elevated. KRT80 is relevant for protein binding, peptide binding, and amide binding and may serve as an important regulator in cell junction and adhesion. (c) A robust correlation between KRT80 and linc01269 (*R* = 0.52, *p* < 0.001) was observed in HNSC samples. These data were acquired from GEPIA2 (http://gepia2.cancer-pku.cn), and the data were obtained from TCGA database. (d) RNA-seq revealed that lncHNSCAT1 was upregulated, which resulted in the upregulation of KRT80. (e) Western blotting confirmed that KRT80 was upregulated after HNSCAT1 was overexpressed in SCL-1 and Cal27 cells. GAPDH served as a control.

To further explore the mechanism underlying the tumor-inhibitory effect of HNSCAT1, RNA-seq analysis was performed in SCL-1 cells after overexpressing lncRNA HNSCAT1 (deposited in NODE database, https://www.biosino.org/node/login, OEZ007550). Through Gene Set Enrichment Analysis (GSEA), we found that cell junction- and adhesion complex-related pathways (KEGG_HSA04530, KEGG_HSA04510, and KEGG_HSA04520) were significantly activated after the restoration of HNSCAT1 expression (Figure 3(a)). In addition, through Circos analysis, we found that keratin 80 (KRT80), which encodes a type II keratin that is involved in terminal epithelial differentiation, was significantly upregulated after overexpression of HNSCAT1 (Figure 3(b)). We further found that in TCGA HNSC samples, KRT80 presented a strong positive correlation with HNSCAT1 (*R* = 0.52, *p* < 0.001, Figure 3(c)). We also further validated our finding in our RNA-seq data. We found that HNSCAT1-overexpressing HNSC cells presented enhanced reads in the KRT80 region (Figure 3(d)) and elevated expression levels at the mRNA (Figure S6) and protein levels (Figure 3(e)). Therefore, KRT80 is a potential downstream target of HNSCAT1.

### 3.4. KRT80 Is Downregulated in HNSC Samples

**Figure 4:**
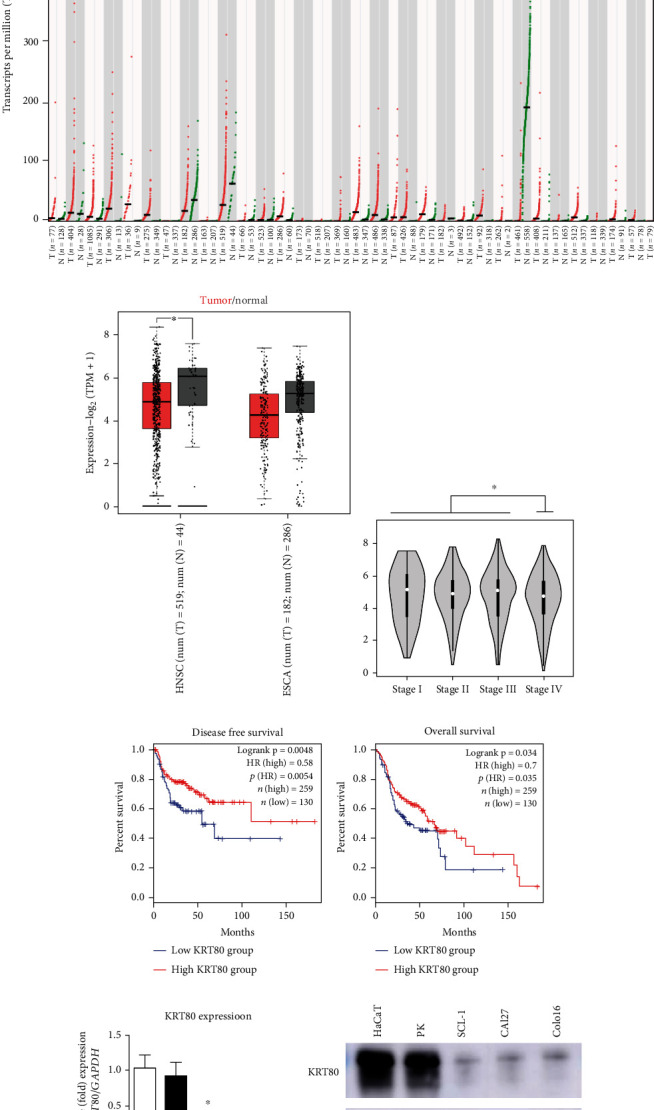
KRT80 is downregulated in HNSC cells. (a) Pancancer analysis of KRT80 RNA expression in TCGA database. (b) Expression of KRT80 in HNSC and normal samples. These data were acquired from GEPIA2 (http://gepia2.cancer-pku.cn). (c) KRT80 presented lower expression in stage IV HNSC samples than in early-stage HNSC samples. (d, e) Elevated KRT80 expression was associated with favorable outcomes in terms of both (d) disease-free survival (log-rank *p* = 0.0048) and (e) overall survival (log-rank *p* = 0.034). (f) Real-time PCR revealed that KRT80 was downregulated in HNSC cell lines. HaCaT cells and primary keratinocytes (PK) served as normal controls. The value of HaCaT cells was set to 1. Experiments were conducted in triplicate, and the results are shown as the mean ± SEM. ^∗^*p* < 0.05. (g) Western blotting assays demonstrated that KRT80 protein was upregulated in HNSC cells (lanes 3, 4, and 5) compared to normal control cells (lanes 1 and 2).

Through a pancancer analysis of TCGA samples (Figure 4(a)), we found that KRT80 presented a similar expression pattern to HNSCAT1: both were significantly downregulated in HNSC and ESCA samples (Figure 4(b)). Moreover, we found that stage IV samples presented with lower expression than early-stage HNSC samples (Figure 4(c)). More importantly, elevated expression of KRT80 was associated with a more favorable outcome in terms of both DFS (Figure 4(d)) and OS (Figure 4(e)). We further validated the expression profiling of KRT80 in our cell lines. As expected, KRT80 was markedly downregulated in HNSC cells at the mRNA (Figure 4(f)) and protein (Figure 4(g)) levels.

### 3.5. Overexpression of KRT80 Attenuates HNSC Malignant Behavior *In Vitro* and *In Vivo*

**Figure 5:**
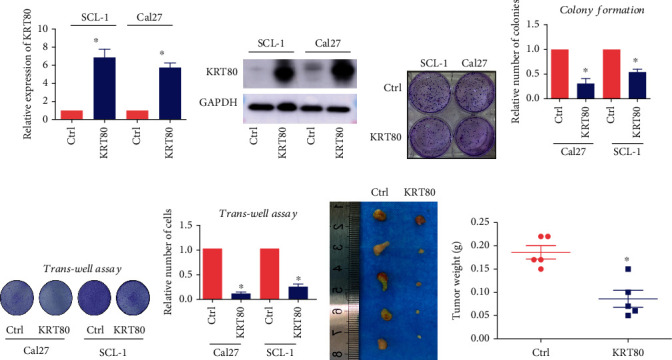
KRT80 serves as a tumor suppressor in HNSC cells. (a) Real-time PCR was conducted to evaluate KRT80 expression after overexpressing KRT80 in SCL-1 and Cal27 cells. (b) Western blotting assays showed that KRT80 protein was increased after overexpression in SCL-1 and Cal27 cells. (c, d) Colony formation assays were conducted to evaluate proliferation capacity after KRT80 overexpression in SCL-1 and Cal27 cells. Experiments were conducted in triplicate, and the results are shown as the mean ± SEM. ^∗^*p* < 0.05. (e, f) Transwell assays showed that migration was impaired in KRT80-overexpressing SCL-1 and Cal27 cells. Experiments were conducted in triplicate, and the results are shown as the mean ± SEM. ^∗^*p* < 0.05. (g, h) Subcutaneous xenografts were established in (g) KRT80-overexpressing and control cells. *N* = 5 for each group. (h) Tumor weight in each xenograft. ^∗^*p* < 0.05.

Since KRT80 presented with decreased expression in HNSC, we overexpressed KRT80 in SCL-1 and Cal27 cells. We found that the mRNA level of KRT80 was significantly upregulated by ~5-fold after overexpression (Figure 5(a)). Consistently, KRT80 protein was significantly enriched in KRT80-overexpressing cells (Figure 5(b)). We were then interested in observing tumor behaviors in these cells. Notably, through a colony formation assay, we found that tumors formed smaller and fewer colonies after overexpression of KRT80 in both SCL-1 and Cal27 cell lines (Figures 5(c) and 5(d)). Moreover, cellular migration was also impaired in KRT80-overexpressing cells (Figures 5(e) and 5(f)). More importantly, we established a subcutaneous xenograft model and found that KRT80-overexpressing SCL-1 cells formed smaller tumors, both in size (Figure 5(g)) and weight (Figure 5(h)). Taken together, these data indicate that both HNSCAT1 and KRT80 are tumor suppressor genes with decreased expression in HNSC.

### 3.6. miR-1254 Interacts with HNSCAT1 and KRT80

**Figure 6:**
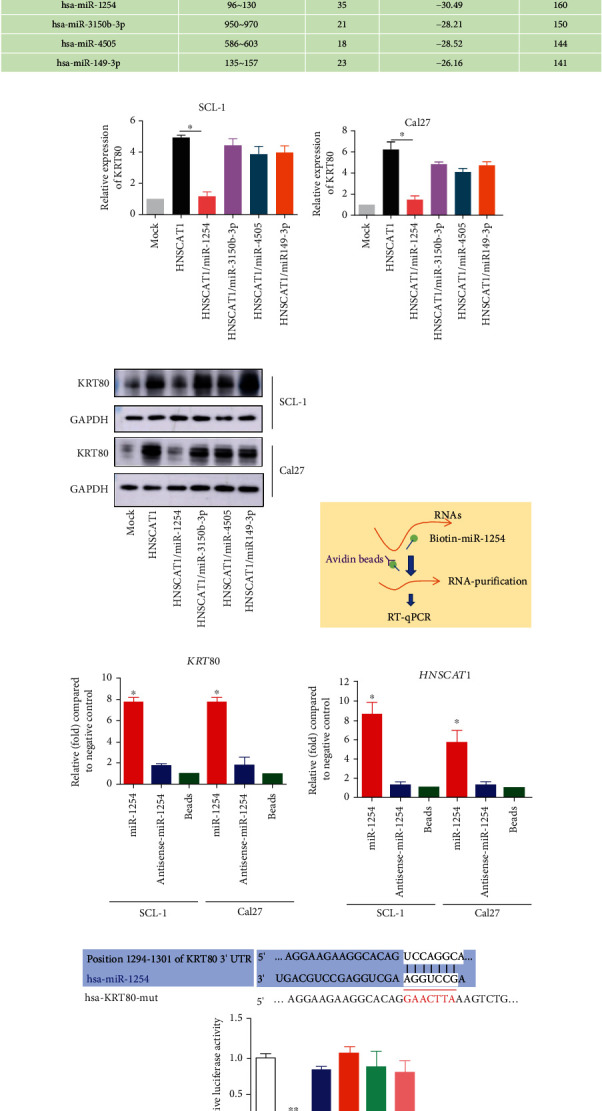
hsa-miR-1254 binds KRT80 and HNSCAT1. (a) Through TargetScan and miRDB, we found 268 miRNAs that may serve as binding candidates for HNSCAT1. Notably, 4 of them (miR-1254, miR-3150b-3p, miR-4505, and miR-149-3p) were predicted to interact with the 3′UTR of KRT80 mRNA. (b, c) Real-time PCR was performed to observe KRT80 expression after transfecting miRNA mimics (miR-1254, miR-3150b-3p, miR-4505, and miR-149-3p) into HNSCAT1-overexpressing cells. Experiments were conducted in triplicate, and the results are shown as the mean ± SEM. ^∗^*p* < 0.05. (d) Western blotting assays were conducted to observe KRT80 protein levels after transfecting miRNA mimics into HNSCAT1-overexpressing cells. (e) Schematic figure of the miRNA-binding RNA capture assay. (f, g) Real-time PCR was performed on these miRNA-binding RNAs. The miRNA probe transfection-free group (beads group) was set to 1. These results indicated that miR-1254 interacts with (f) KRT80 and (g) lincRNA HNSCAT1. (h) A reporter gene assay demonstrated that miR-1254 mimics significantly inhibited reporter activity, while the miRNA potential binding site-mutated group (lanes 4-6) and miR-1254 inhibitor-treated group (lane 3) presented similar reporter signals compared to the control group. Experiments were conducted in triplicate, and the results are shown as the mean ± SEM. ^∗^*p* < 0.05.

We were then curious about the molecular basis of the mechanism of the regulation of lncRNA HNSCAT1 and KRT80. Because lncHNSCAT1 is a cytoplasmic transcript, we hypothesized that lncRNA HNSCAT1 mainly functions through a competing endogenous RNA (ceRNA) network. First, we investigated potential HNSCAT1-binding miRNAs. Through TargetScan and miRDB, we found 268 miRNAs that may serve as binding candidates for HNSCAT1. Notably, 4 of them (miR-1254, miR-3150b-3p, miR-4505, and miR-149-3p) could also potentially interact with the 3′UTR of KRT80 mRNA (Figure 6(a)). To further investigate which miRNA served as the regulator of HNSCAT1/KRT80, we transfected these miRNA mimics into HNSCAT1-overexpressing HNSC cells. Here, we found that miR-1254 significantly inhibited the expression of KRT80, while KRT80 expression remained unchanged in other miRNA groups (Figures 6(b) and 6(c), Figure S7). Consistently, the KRT80 protein signal also showed a significant reduction after exogenous miR-1254 transfection (Figure 6(d)). More importantly, we then established biotin-labeled miRNA-1254 and used it as a bait to capture miR-1254-interacting mRNAs (Figure 6(e)). Through a biotin pull-down assay, we identified that miR-1254 interacts with both KRT80 and HNSCAT1 mRNA, whereas the antisense miRNA showed a signal intensity similar to that of the negative control (only streptavidin beads) group (Figures 6(f) and 6(g)). Moreover, miRNA inhibition potencies were assessed by the dual-luciferase assay, whereby one of the reporter genes encoding Renilla luciferase was fused to wild-type and mutated 3′UTR of KRT80. Here, we found that miR-1254 mimics significantly inhibited reporter activity, while the miRNA potential binding site-mutated group (lane 4-6) and miR-1254 inhibitor-treated group (lane 3) presented reporter signals similar to those of the control group (Figure 6(h)). These data indicated that miR-1254 could bind with lncHNSCAT1 and KRT80 and inhibit KRT80 expression by interacting with its 3′UTR.

### 3.7. miR-1254 Modulates HNSC Behaviors by Inhibiting KRT80

**Figure 7:**
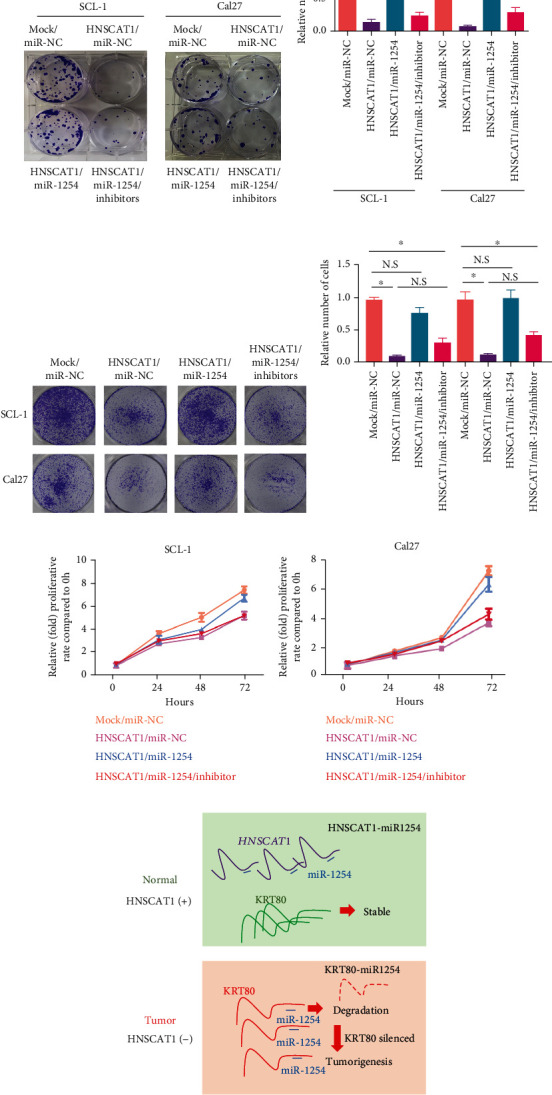
miR-1254 promotes HNSC malignant behavior by inhibiting KRT80. (a, b) Colony formation assays were performed to determine proliferation capacity after overexpressing miRNA-1254 in SCL-1 and Cal27 cells. Experiments were conducted in triplicate, and the results are shown as the mean ± SEM. ^∗^*p* < 0.05. (c, d) Transwell assays showed that the migration ability was impaired after overexpressing miRNA-1254 in SCL-1 and Cal27 cells. All of the experiments were performed in triplicate and are presented as the mean ± SEM. ^∗^*p* < 0.05. (e, f) The CCK-8 assay indicated that the cell growth inhibition effect after HNSCAT1 overexpression was largely rescued when miR-1254 was reintroduced; however, the rescue efficacy was diminished after the addition of extra miR-1254 inhibitors in both (e) SCL-1 and (f) Cal27 cells. (g) Proposed mechanism of the lncRNA HNSCAT1/miR-1254/KRT80 axis in HNSC. HNSCAT1 could interact with miR-1254 and thereby prevent miR-1254-mediated KRT80 degradation. lncHNSCAT1 and KRT80 serve as important tumor suppressors in HNSC.

Since miR-1254 bridges the regulation of HNSCAT1 and KRT80 expression in HNSC cells, we were interested in assessing the role of the HNSCAT1/miR-1254/KRT80 axis in HNSC tumorigenesis. In the colony formation assay, we found that HNSCAT1 overexpression-mediated tumor inhibition was largely compromised after introducing exogenous miR-1254 (Figures 7(a) and 7(b), lane 3), but the inhibitory effect could be rescued by adding miR-1254 inhibitors (Figures 7(a) and 7(b), lane 3). Moreover, for cell migration analysis, we found that miR-1254 could also attenuate the effect of HNSCAT1 overexpression. Importantly, this inhibitory phenomenon could also be neutralized by miR-1254 inhibitors (Figures 7(c) and 7(d)). For cell growth analysis, we found that the cell growth inhibition effect after HNSCAT1 overexpression was largely restored when miR-1254 was reintroduced; however, the rescue efficacy was diminished after treatment with additional miR-1254 inhibitors in both SCL-1 (Figure 7(e)) and Cal27 cells (Figure 7(f)). Thus, these results indicate that miR-1245 bridges the regulatory relationship between KRT80 and lncHNSCAT1.

## 4. Discussion

Globally, HNSC is the 6^th^ most prevalent human malignancy, accounting for more than 380 thousand mortalities annually worldwide [6, 19, 24]. In recent decades, the complexity and heterogeneity of HNSC have been recognized [25]. In parallel, the development of high-throughput omics has given rise to a better picture of the behavior and characteristics of molecules during carcinogenesis [26]. lncRNAs are a class of functional RNA molecules that do not have translational capacities but play a vital role in the activities of transcription factors or regulate structural changes in chromatin. However, the function of lncRNAs in HNSC needs to be further explored.

To date, several lncRNAs have been recognized to have vital roles in cancer progression as well as metastasis [27–31]. For instance, HOTAIR is upregulated in HNSC, and its upregulation is related to an elevated rate of metastasis. More specifically, HOTAIR triggers the epithelial-mesenchymal transition (EMT) process by recruiting EZH2 (an H3K27me3 modifier enzyme), which has been proven to be negatively associated with clinical outcomes in HNSC patients [32, 33]. Most importantly, upregulation of the long noncoding RNA EGFR-AS1 mediates epidermal growth factor receptor addiction and modulates treatment resistance toward tyrosine kinase inhibitors (TKIs) in squamous cell carcinoma. EGFR-AS1 downregulation shifts splicing toward EGFR isoform D, leading to ligand-mediated pathway activation [22]. Herein, we found that lncRNA linc01269 was one of the most downregulated lncRNAs in HNSC versus normal samples. Through RACE analysis, we found two linc01269-related transcripts with different 5′ and 3′ boundaries, presenting a novel transcript, which we named *HNSC-associated transcript 1* (*HNSCAT1*). Moreover, overexpression of HNSCAT1 significantly inhibited tumor progression through HNSCAT1 interaction with miR-1254 and rescued KRT80 expression. Our study is the first to report existence and function of a novel transcript, HNSCAT1.

miRNA aberrations have been frequently identified in a wide variety of cancers [34]. For HNSC, previous studies have found 128 miRNAs were differentially expressed in HNSCC tissue in TCGA dataset. Moreover, several individual miRNAs have shown indicative regulatory functions [35]. For instance, miR-204-5p suppresses epithelial-mesenchymal transition (EMT) and STAT3 signaling by targeting SNAI2, SUZ12, HDAC1, and JAK2 [36]. In addition, HPV+ HNSCC-derived exosomal miR-9 induces macrophage M1 polarization and increases tumor radiosensitivity, which provides a novel miRNA-based therapy [37]. However, the function of miR-01254 remains unexplored in HNSC. Herein, we found for the first time that miR-1254 interacts with both lncRNA HNSCAT1 and KRT80. After overexpression of HNSCAT1, the inhibition of miR-1254-induced KRT80 degradation was partially rescued. The HNSCAT1/miR-1254/KRT80 signaling pathway serves as an important regulator in HNSC, and targeted treatments that address these epigenetic deficiencies might be promising strategies for HNSC.

Keratins have been revealed to play a vital role in the tumorigenesis of numerous cancer types [38–40]. In HNSC, highly keratinized squamous cell carcinoma exhibits an improved response to treatment and prognosis compared with weakly keratinized cancer [41]. Herein, we found that an important keratin, keratin 80, was significantly downregulated in HNSC. Although KRT80 has been reported to play an oncogenic role in colorectal carcinoma and endocrine-resistant breast cancer [42], the role of KRT80 in HNSC remains unexplored. Here, for the first time, we identified KRT80 as a biomarker of a favorable outcome in HNSC; thus, KRT80 overexpression may serve as an alternative strategy for HNSC treatment.

In this study, we identified a novel cytoplasmic transcript, HNSC-associated transcript 1 (HNSCAT1, previously recognized as linc01269), that was downregulated in tumor samples and was also associated with favorable outcomes in HNSC. lncHNSCAT1 serves as a necessary tumor suppressor, and overexpression of lncHNSCAT1 triggered therapeutic efficacy in HNSC both *in vitro* and *in vivo*. More importantly, we identified KRT80 as the target of HNSCAT1. KRT80 expression was modulated by lncRNA HNSCAT1 and presented a positive correlation in TCGA cohort. Intriguingly, we then identified that miR-1245 could simultaneously interact with KRT80 and HNSCAT1, connecting the regulatory relationship between KRT80 and HNSCAT1. Conclusively, our study demonstrated that the lncRNA HNSCAT1/miR-1245/KRT80 axis functions as a necessary tumor-suppressive signaling pathway in HNSC, which highlights a novel mechanism of lncRNA function and provides alternative targets for the diagnosis and treatment of HNSC.

## Data Availability

RNA-seq analysis was performed in SCL-1 cell after overexpressing lncRNA HNSCAT1 (deposited in NODE database, https://www.biosino.org/node/login, OEZ007550). Raw images of the Western blot assay have been displayed in the supplementary figures.

## Abbreviations

lncRNAs: Long noncoding RNAs
HNSC: Head and neck squamous carcinoma
HNSCAT1: HNSC-associated transcript 1
KRT80: Keratin 80
NODE: The National Omics Data Encyclopedia
OS: Overall survival
DFS: Disease-free survival
RACE: Rapid amplification of cDNA ends
PK: Primary keratinocyte.

## Supplementary Materials

Supplementary Materials Supplementary figures and legends of this manuscript.
