# Supplementary material for: The Long Noncoding Transcript HNSCAT1 Activates KRT80 and Triggers Therapeutic Efficacy in Head and Neck Squamous Cell Carcinoma
Source: Oxid Med Cell Longev. 2022 Aug 4;2022:4156966. doi: 10.1155/2022/4156966 (PMC9371835; doi:10.1155/2022/4156966)
Supplement: Supplementary Materials — Supplementary figures and legends of this manuscript. [file 4156966.f1.docx]

**Supplementary Figures and legends**


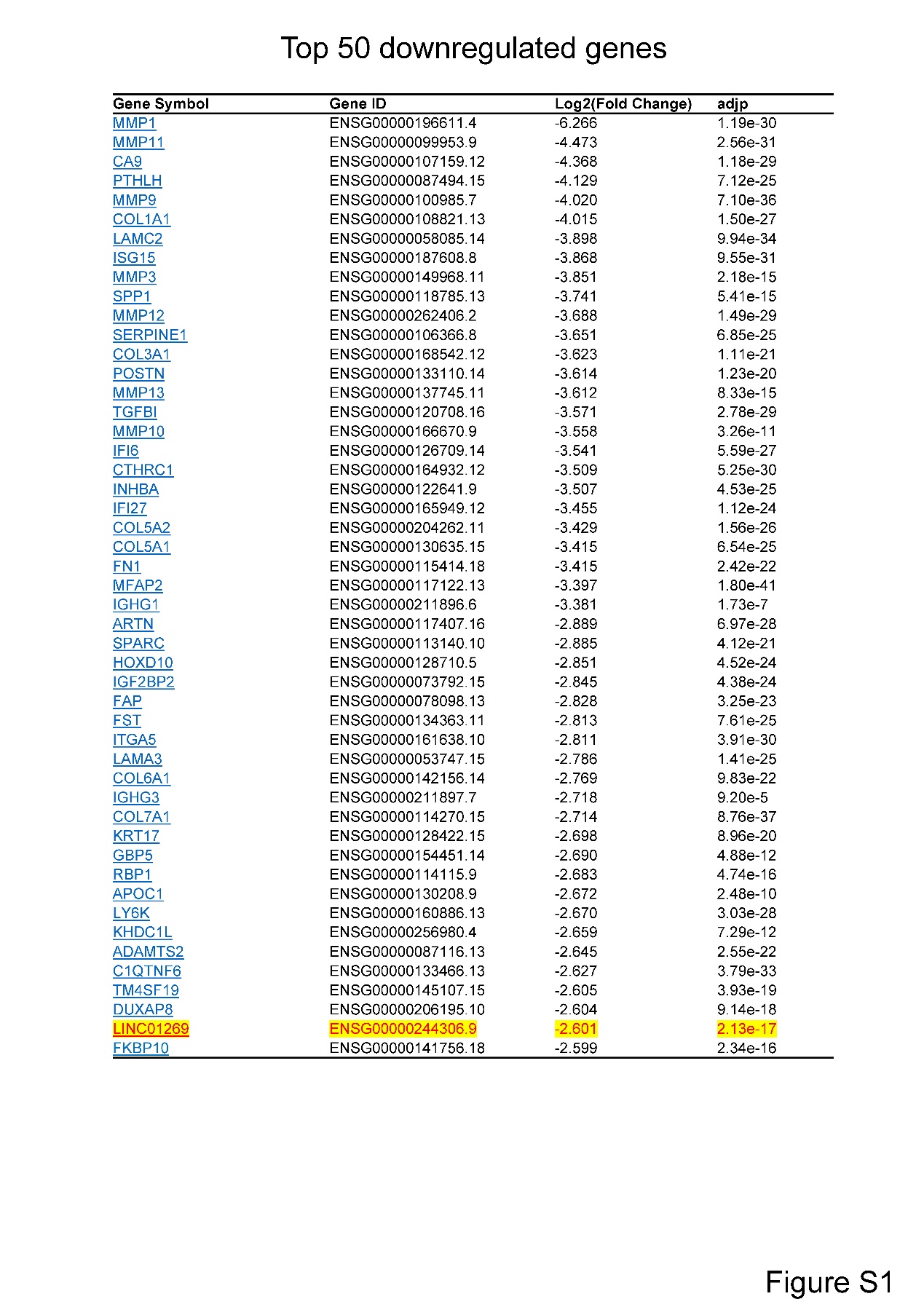


**Figure S1:** Transcripts that are downregulated in HNSC in TCGA cohort. The data was acquired in GEPIA database (http://gepia.cancer-pku.cn/).


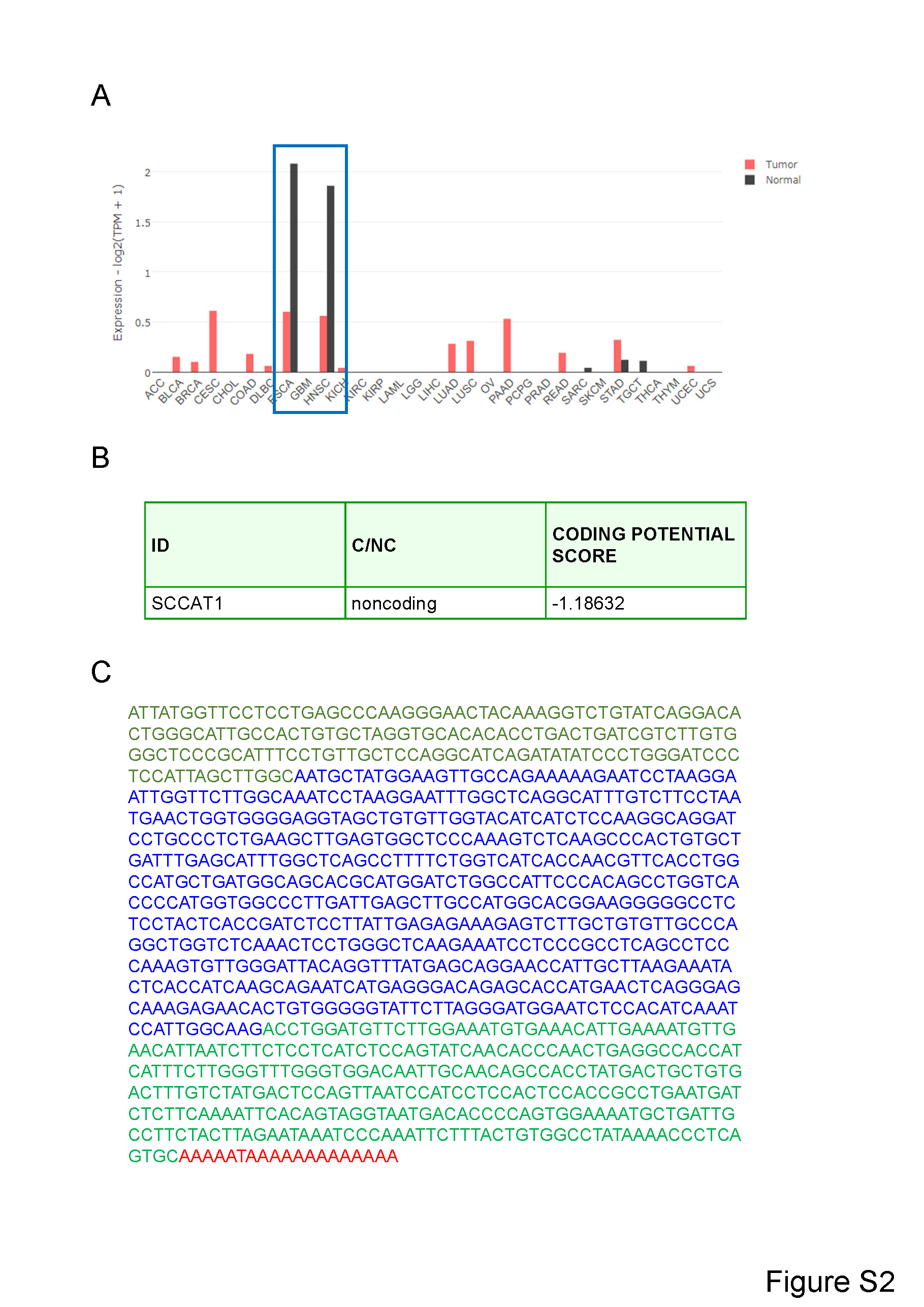
**Figure S2:** Characteristics of lncRNA HNSCAT1.

(A) Pan-cancer analysis of linc01269 in TCGA cohort. Red column represents tumor samples and black column indicates its normal control.

(B) Coding potential of linc01269.

(C) Full length of HNSCAT1, revealed by 5’RACE and 3’RACE assay.

**
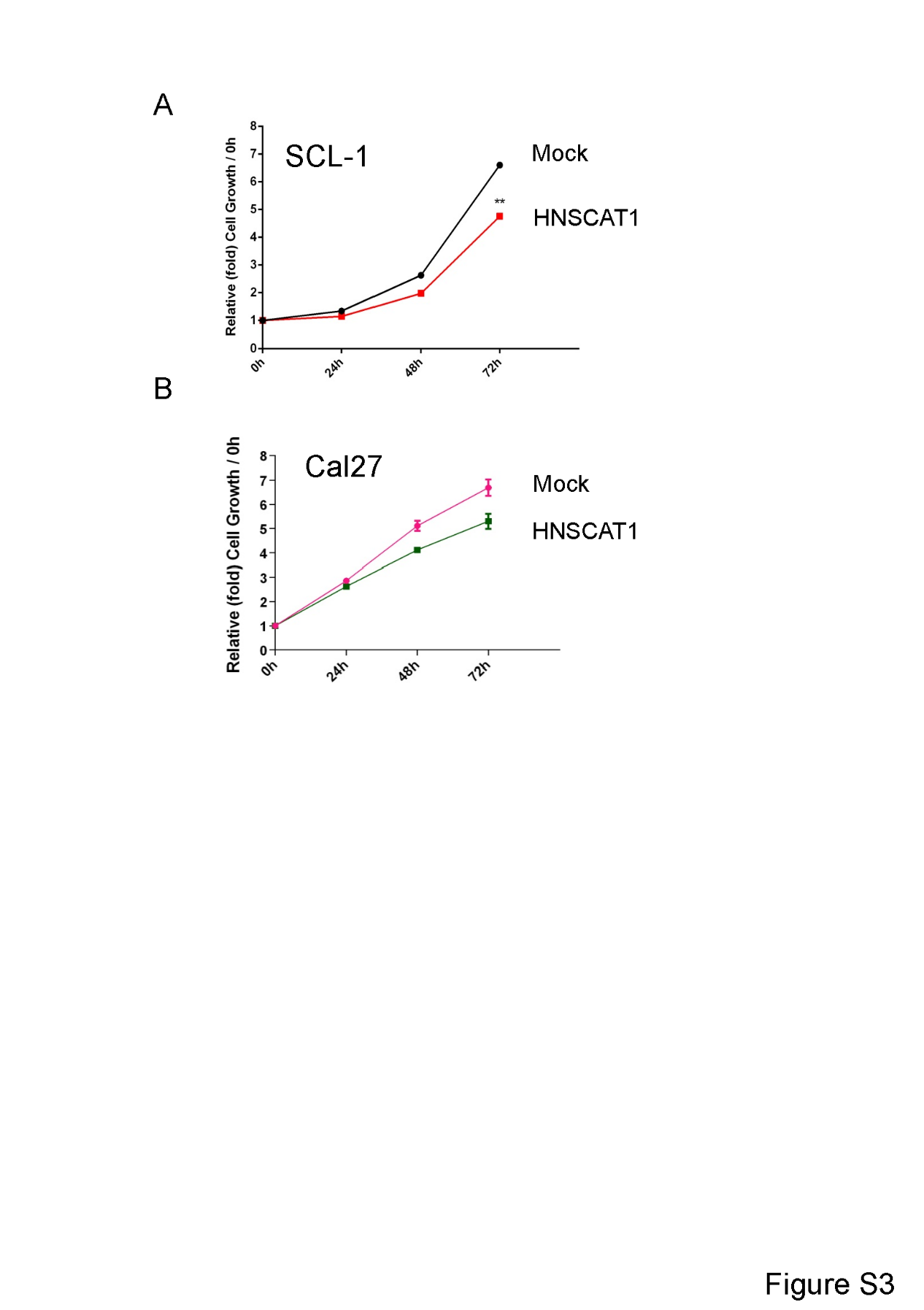
Figure S3:** Overexpression of HNSCAT1 attenuate HNSC growth.

(A-B) CCK-8 assay in SCL-1 and Cal27 after overexpressing lncRNA HNSCAT1. The black line indicates control group and the red line refers to HNSCAT1 overexpressed group. This data has been replicated three times and representative result is shown. **p<0.01.


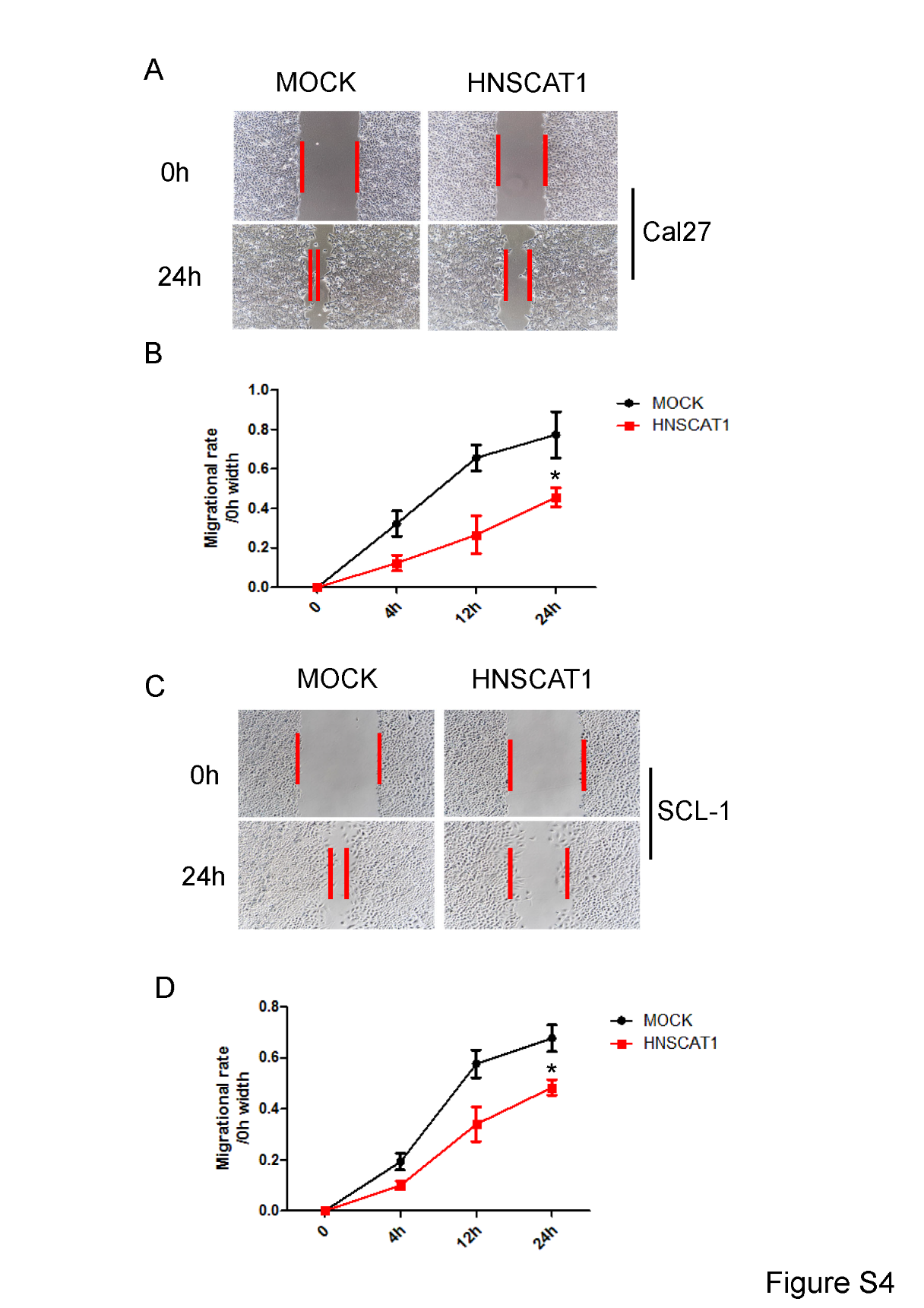


**Figure S4:** Overexpression of HNSCAT1 attenuate HNSC migration.

Scratch assay was performed to evaluate cellular migration ability after overexpressing lncRNA HNSCAT1 in. Cal27(A-B) and SCL-1 (C-D). The black line indicates control group, and the red line refers to HNSCAT1 overexpressed group. This data has been replicated three times. *p<0.05.


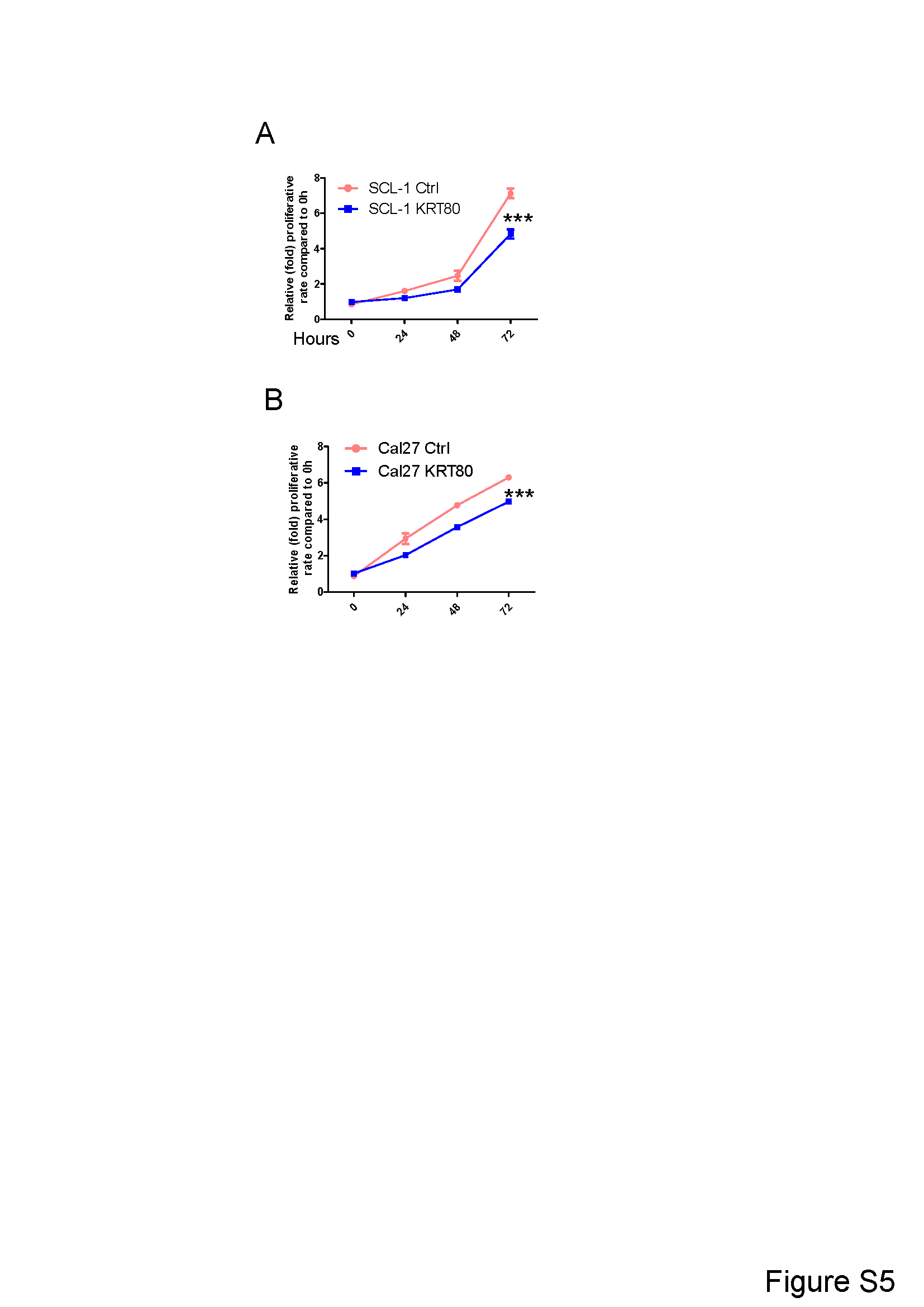


**Figure S5:** Overexpression of KRT80 attenuate HNSC growth.

(A-B) CCK-8 assay in SCL-1 and Cal27 after overexpressing lncRNA HNSCAT1. The red line indicates control group, and the blue line refers to KRT80 overexpressed group. This data has been replicated three times and representative result is shown. ***p<0.001.


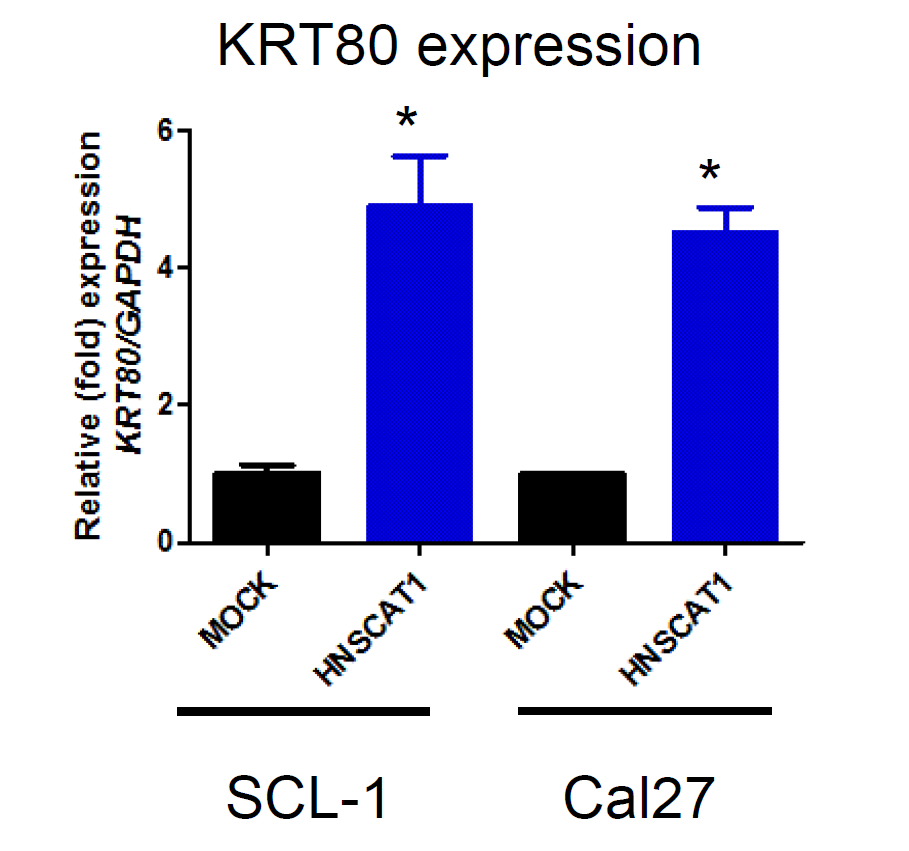


**Figure S6:** Realtime PCR revealed KRT80 expression was increased after overexpressing HNSCAT1. Red column represents HNSCAT1 overexpressed group and black column indicates its normal control.


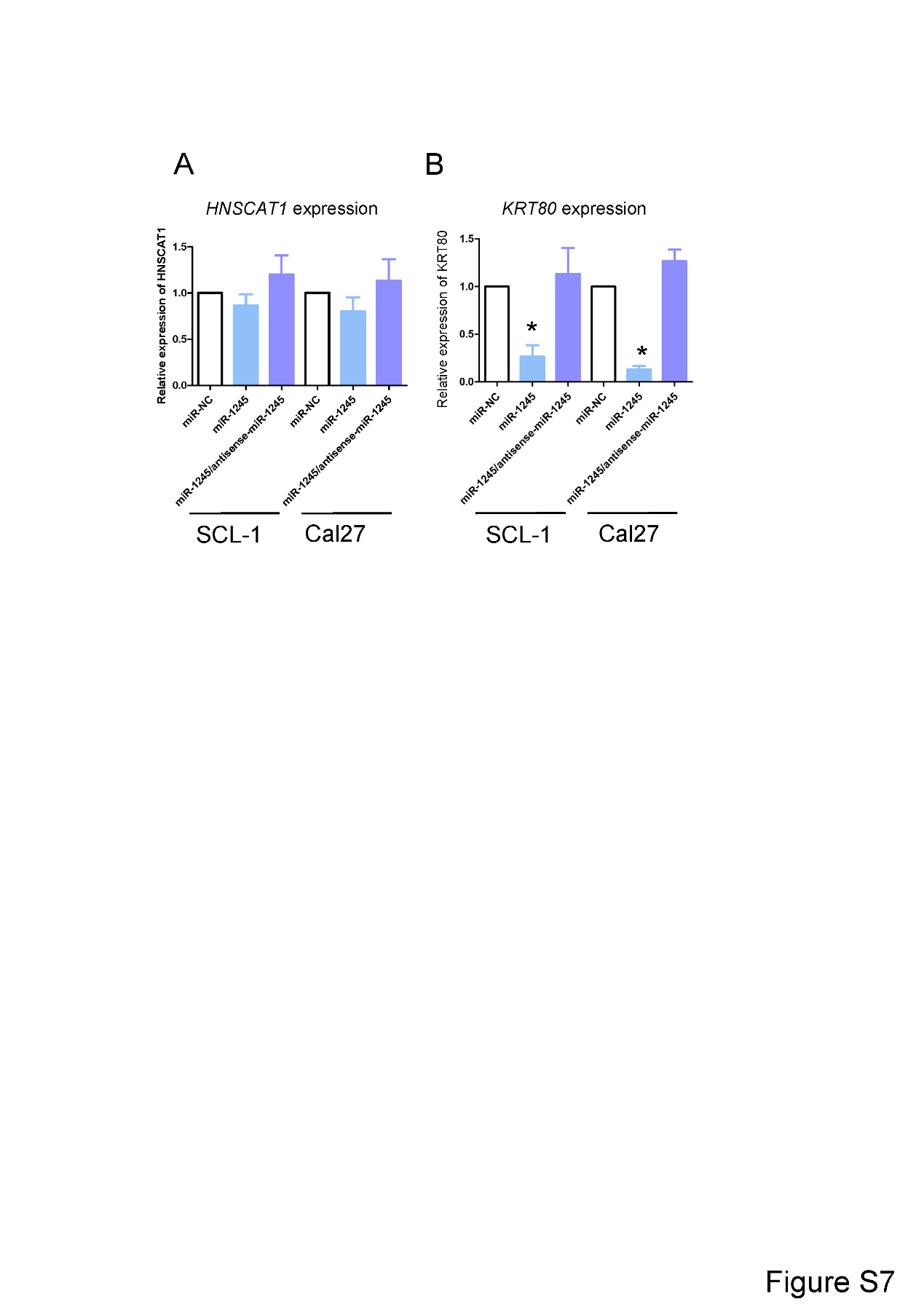
**Figure S7:** Realtime PCR revealed KRT80 expression was increased after overexpressing miR1245 while HNSCAT1 remain unchanged upon its binding. *p<0.05.


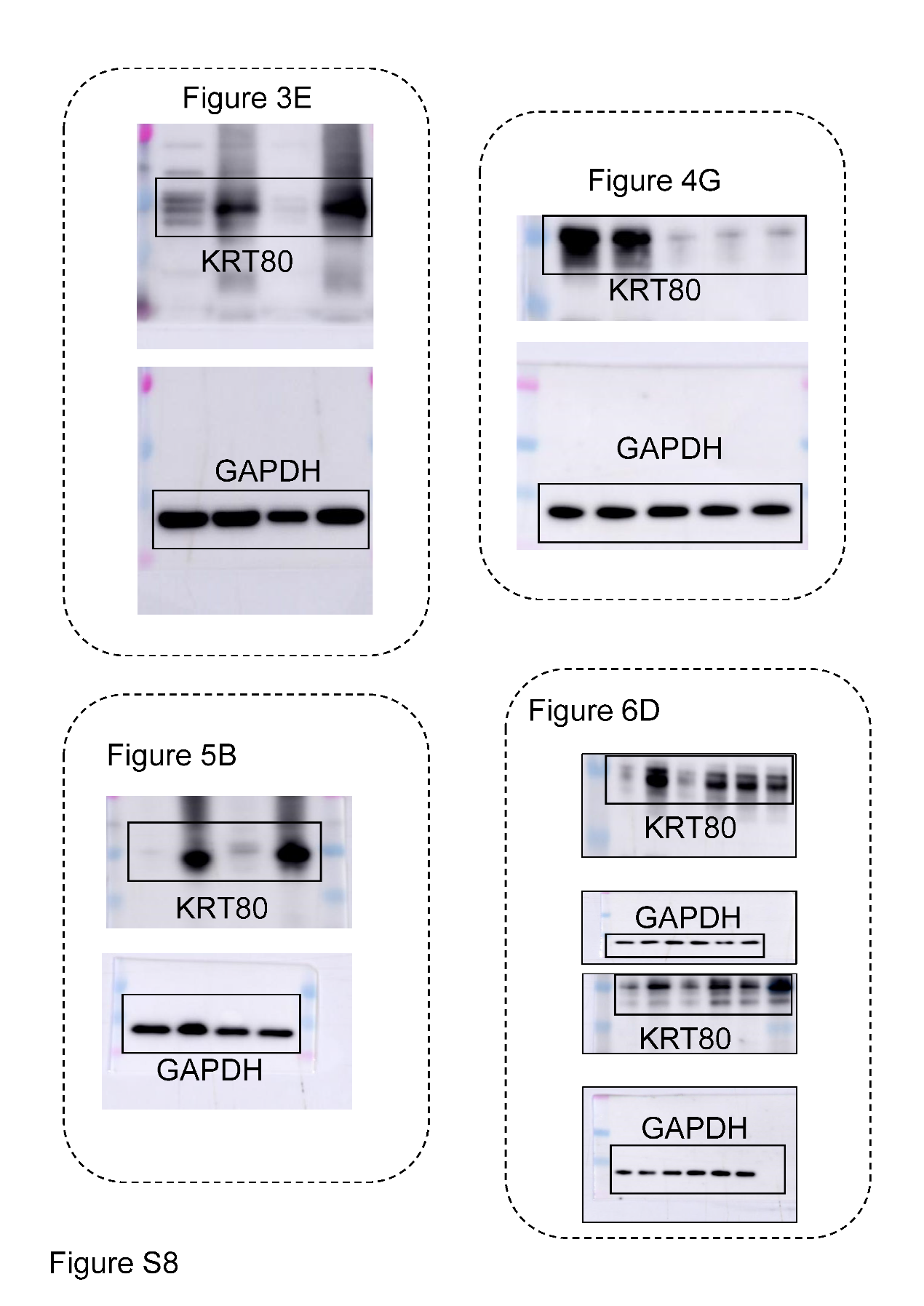


**Figure S8:** Raw images of western blot in this study.
